# Modification of Diet in Renal Disease (MDRD) Study and CKD Epidemiology Collaboration (CKD-EPI) Equations for Taiwanese Adults

**DOI:** 10.1371/journal.pone.0099645

**Published:** 2014-06-13

**Authors:** Ling-I Chen, Jinn-Yuh Guh, Kwan-Dun Wu, Yung-Ming Chen, Mei-Chuan Kuo, Shang-Jyh Hwang, Tzu-Hui Chen, Hung-Chun Chen

**Affiliations:** 1 Division of Nephrology, Department of Internal Medicine, Kaohsiung Medical University Hospital, Kaohsiung, Taiwan; 2 Department of Nursing, Kaohsiung Medical University Hospital, Kaohsiung, Taiwan; 3 Department of Internal Medicine, National Taiwan University Hospital, Taipei, Taiwan; 4 Faculty of Renal Care, College of Medicine, Kaohsiung Medical University, Kaohsiung, Taiwan; INSERM, France

## Abstract

**Background:**

Estimated glomerular filtration rate (eGFR) using the Modification of Diet in Renal Disease (MDRD) study or the Chronic Kidney Disease Epidemiology Collaboration (CKD-EPI) equations may not be accurate for Asians; thus, we developed modified eGFR equations for Taiwanese adults.

**Methods:**

This cross-sectional study compared the Taiwanese eGFR equations, the MDRD study, and the CKD-EPI equations with inulin clearance (C_in_). A total of 695 adults including 259 healthy volunteers and 436 CKD patients were recruited. Participants from the Kaohsiung Medical University Hospital were used as the development set (N = 556) to develop the Taiwanese eGFR equations, whereas participants from the National Taiwan University Hospital were used as the validation set (N = 139) for external validation.

**Results:**

The Taiwanese eGFR equations were developed by using the extended Bland-Altman plot in the development set. The Taiwanese MDRD equation was 1.309×MDRD^0.912^, Taiwanese CKD-EPI was 1.262×CKD-EPI^0.914^ and Taiwanese four-level CKD-EPI was 1.205×four-level CKD-EPI^0.914^. In the validation set, the Taiwanese equations had the lowest bias, the Taiwanese equations and the Japanese CKD-EPI equation had the lowest RMSE, whereas the Taiwanese and the Japanese equations had the best precision and the highest P_30_ among all equations. However, the Taiwanese MDRD equation had higher concordance correlation than did the Taiwanese CKD-EPI, the Taiwanese four-level CKD-EPI and the Japanese equations. Moreover, only the Taiwanese equations had no proportional bias among all of the equations. Finally, the Taiwanese MDRD equation had the best diagnostic performance in terms of ordinal logistic regression among all of the equations.

**Conclusion:**

The Taiwanese MDRD equation is better than the MDRD, CKD-EPI, Japanese, Asian, Thai, Taiwanese CKD-EPI, and Taiwanese four-level CKD-EPI equations for Taiwanese adults.

## Introduction

The abbreviated Modification of Diet in Renal Disease (MDRD) study equation [1] was derived from Caucasians and African Americans with chronic kidney diseases (CKD) [2] and is not accurate for Asians [3]–[6] or when the estimating equations for glomerular filtration rate (eGFR) are above 60 mL/min/1.73 m^2^ [7]. Thus, some Asian countries have developed their own eGFR equations [5], [8]–[11]. However, many equations were derived solely from CKD patients, thereby having limitations in application to the general population [12], [13]. For example, the MDRD equation underestimated the gold standard GFR measured by inulin clearance (C_in_) for those with C_in_ of greater than 60 mL/min/1.73 m^2^ in a recent Japanese study [4].

The Chronic Kidney Disease-Epidemiology Collaboration (CKD-EPI) equation may be more accurate than the MDRD Study equation, particularly at higher levels of GFR and in populations without CKD [14], [15]. Thus, the Kidney Disease Improving Global Outcomes (KDIGO) 2012 guidelines recommend using the CKD-EPI equation in adults unless an alternative equation has been shown to be more accurate in the local population [16]. Additionally, the four-level CKD-EPI equation (Black, Asian, Native American and Hispanic, White and other) may improve accuracy for Asians [17].

Taiwan has a high prevalence (11.93%) and a low awareness (3.54%) of CKD [18]. In order to diagnose CKD by an eGFR equation with better accuracy based on the native data, we used C_in_ as the gold standard to develop modified Taiwanese eGFR equations from a cohort of CKD and healthy people and compared the performance between the original and the modified MDRD Study and the CKD-EPI equations.

## Materials and Methods

We recruited adults aged over 18 years to sign informed consents from the Kaohsiung Medical University Hospital and the National Taiwan University Hospital. Subjects with acute renal failure, allergy to inulin, pregnancy, problems in voiding, amputation, congestive heart failure, cirrhosis with ascites, use of cimetidine or trimethoprim, oliguria, and those who had ever received any renal replacement therapy were excluded. Healthy volunteers were enrolled according to the percentage of age distribution in Taiwanese reported by the Ministry of the Interior of Taiwan. CKD was diagnosed and classified according to the K/DOQI clinical guidelines [1]. The ratio of the number of the CKD patients to healthy volunteers was approximately 2∶1 in this study.

### Ethics Statement

The study protocol was approved by the Institutional Review Board of the Kaohsiung Medical University Hospital (KMUH-IRB-960304) and National Taiwan University Hospital (NTUH-IRB-201002031M). Informed consents were obtained in written form from patients and all clinical investigations were conducted according to the principles expressed in the Declaration of Helsinki. The patients gave consent for the publication of the clinical details.

### Inulin clearance

All subjects underwent procedures to measure C_in_ at the Kaohsiung Medical University Hospital or the National Taiwan University Hospital with the same protocol after the approval by the Ethics Committee in each respective hospital. C_in_ was calculated from serum inulin, urine inulin concentration, and urine volume collected in each time period. The protocol has been described before [9], [19]. Briefly, after overnight fasting, the subject drank 500 mL of water 30 minutes before intravenous injection of inulin (40 mL 10% inulin in 360 mL 0.54% NaC1 with a final concentration of 1%, Fuji Yakuhin Co. Ltd., Saitama, Japan). Just before the infusion, complete urine collection and blood sampling were performed in an ordinary way. To maintain hydration, 60 mL of water was drunk at 30 and 60 minutes after the start of inulin infusion. The rate of inulin infusion was 300 mL/hour for the first 30 min and 100 mL/hour for the following 60 min.

Blood samples for serum inulin concentration were collected at 45 min and 75 min after the start of inulin infusion. Urine samples for urinary inulin concentration were collected between 30 and 60 min and between 60 and 90 min after the patient completely voided the bladder at 30 min. The first and second urinary excretions of inulin were added, assuming the total amount as a single urine collection between 30 and 90 min [9]. This value was then used with the mean of the first (45 minutes) and second (75 minutes) serum inulin concentrations to calculate C_in_ as the measured GFR (mL/min/1.73 m^2^) [9]. Note that this abbreviated method for the calculation of C_in_ has been shown to be equivalent to the full 2-hour C_in_ protocol [9].

Serum inulin was measured by an enzymatic method by using a commercial kit (Diacolor Inulin; Toyobo Co, Osaka, Japan) with Hitachi 7180 auto-analyzer at the Kaohsiung Medical University Hospital. The steady-state of serum inulin concentrations was reached at 45 min (22.2 [21.5, 22.9] mg/dL) and 75 min (21.6 [21.1, 22.1] mg/dL) because the coefficient of variation was only 2.9%. This result was similar to a previous study [9].

### Measurement of creatinine and estimated GFR

Serum creatinine (SCr) was measured by the IDMS-traceable enzymatic method in a Roche Cobas Integra 400 at the Kaohsiung Medical University Hospital. The eGFR values were calculated by the IDMS-traceable MDRD equation: 175×SCr^-1.154^×Age^-0.203^×0.742 (if female), the CKD-EPI equation: 141×min(SCr/κ, 1)^α^ ×max(SCr/κ, 1)^-1.209^×0.993^Age^×1.018 [if female] where κ is 0.7 for females and 0.9 for males, α is −0.329 for females and −0.411 for males, min indicates the minimum of SCr/κ or 1, and max indicates the maximum of SCr/κ or 1, and the four-level CKD-EPI equation [17]: 141×min(SCr/κ, 1)^α^×max(SCr/κ, 1)^−1.210^×0.993^Age^×0.993 [if female]×1.05 [if Asian] where κ is 0.7 for females and 0.9 for males, α is −0.328 for females and −0.412 for males, min indicates the minimum of SCr/κ or 1, and max indicates the maximum of SCr/κ or 1.

Additionally, the Japanese modifications of MDRD ( = 0.808×MDRD) and CKD-EPI ( = 0.813×CKD-EPI) [10], Asian (Chinese, Malays and Indians in Singapore) modifications of MDRD ( = 1.086×MDRD) and CKD-EPI ( = 1.049×CKD-EPI) [11] and Thai modification of MDRD ( = 1.129×MDRD) [20] were calculated.

### Statistical analysis

Continuous data were expressed as the mean ± standard error of the mean or mean (95% confidence interval) unless stated otherwise. Linear regression was expressed as the prediction equation ± standard deviation of prediction. The 95% prediction interval was calculated as the estimate ±2 standard deviations of prediction. Note that the 95% prediction interval is always wider than the 95% confidence interval because the former estimates the scatter of the data whereas the latter estimates only the mean [21]. Continuous variables were compared by unpaired *t*-tests whereas categorical variables were compared by χ^2^ tests unless stated otherwise. All eGFR equations were compared to the Taiwanese MDRD equation.

The Taiwanese equations were generated from the whole development set by using linear regression of the difference on the average (i.e. the extended Bland-Altman plot in the MethComp package in R, which was designed specifically for method comparison studies) [22] of the log-transformed C_in_ and the MDRD, CKD-EPI and four-level CKD-EPI equations.

Internal validation of the Taiwanese equations was performed by 2,000 bootstraps in which the subjects were drawn at random with replacement [23]. Hence, subjects may be represented zero or many times in a bootstrap sample. The Taiwanese equations were trained in the sampled subjects and tested in the original development set in each bootstrap step. Afterwards, the difference of RMSE between the bootstrap and the test samples was subtracted from the RMSE of the original development set [23].

For the validation set, eGFR equations were assessed for accuracy and agreement. Accuracy was measured as the percentage within 30% of C_in_ (P_30_), bias, precision, and the root mean squared error (RMSE) [24]. The bias was defined as the median difference between C_in_ and eGFR (C_in_ - eGFR) with negative values indicating overestimation of C_in_. Precision was expressed as the interquartile range of the bias. RMSE was defined as the square root of the average squared difference of C_in_ and eGFR. RMSE was compared by using 2,000 bootstrap samples to derive the standard errors of the differences of RMSE [11]. Because P_30_ and bias were compared between the paired data, P_30_ was compared by the exact McNemar test, whereas bias was compared by the Wilcoxon signed rank test.

Agreement between two continuous variables (C_in_ and eGFR) was measured by the concordance correlation coefficient (r) [25] and the Bland-Altman plot [22], [26]. Concordance correlation was compared by Zou's method [27]. Note that the comparison of two r's (r of C_in_ and eGFR1 and r of C_in_ and eGFR2) requires a third r (r of eGFR1 and eGFR2). Diagnostic performance of the eGFR equations for the classification of CKD stages (according to C_in_ or eGFR alone, disregarding proteinuria) was assessed by ordinal logistic (generalized ordered logit) regression and chance-corrected agreement (kappa coefficient, a measure of the agreement between two categorical variables) [28]. Ordinal logistic regressions (C_in_-defined CKD category and eGFR was the dependent and independent variable, respectively) (Methods S1) were compared by the Akaike information criterion (AIC) where a model with the lowest AIC is the best model [29]. Note that the AIC is an information-theoretic approach in which there are no null hypothesis significance tests to choose the lowest AIC [29]. Instead, the Akaike weight (*wi*) is used to weigh the evidence of each model [29], [30].
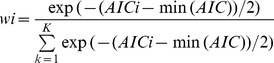



Here, exp denotes the exponential, *AICi* denotes the *AIC* for the *i*th model whereas min (*AIC*) denotes the minimal *AIC* for the *K* models.

Analyses were computed by using the R (version 3.0.0; Free Software Foundation, Boston, MA) and the Stata software (version 13.0, StataCorp LP, College Station, TX).

## Results

### Characteristics of the Participants in the Development and Validation Sets

From April, 2008 to October, 2009, a total of 300 persons were recruited from the Kaohsiung Medical University Hospital where 11 subjects were excluded due to incomplete data. From Oct. 2009 to June 2011, a total of 406 persons were recruited from either the Kaohsiung Medical University Hospital or the National Taiwan University Hospital. Thus, a total of 695 participants which included 259 healthy volunteers and 436 CKD patients were recruited.

Participants from the Kaohsiung Medical University Hospital (located in the southern Taiwan) were used as the development set (N = 556) to develop the Taiwanese eGFR equations whereas participants from the National Taiwan University Hospital were used as the validation set (N = 139) for the external validation of the Taiwanese eGFR equations. Note that this was a geographical external validation set [31], in that the National Taiwan University Hospital is a different hospital located in Northern Taiwan. As shown in Table 1, there were no healthy volunteers, and the participants were older in the validation set.

**Table 1:** Clinical characteristics of the participants.

Clinical characteristics	Development set (n = 556)	Validation set (n = 139)	P
Age (years)	47±0.7	51±1	0.006
Men (%)	47.1	51	0.40
Height (cm)	163±0.4	163±0.8	0.96
Weight (kg)	64±0.5	62±1	0.14
Body surface area (m^2^)	1.69±0.01	1.67±0.01	0.28
CKD clinic patient (%)	53.4	100.0	<0.001
Diabetes mellitus (%)	12.0	12.2	0.95
Serum creatinine (mg/dL)	1.52±0.05	1.43±0.1	0.45
C_in_ (mL/min/1.73 m^2^)	67±1.6	68.8±3.0	0.56
C_in_<60 mL/min/1.73 m^2^ (%)	43.5	42.5	0.82

Abbreviations: CKD, chronic kidney disease; C_in_, inulin clearance.

### Determination and bootstrap cross-validation of the Taiwanese eGFR equations in the development set

**Figure 1:**
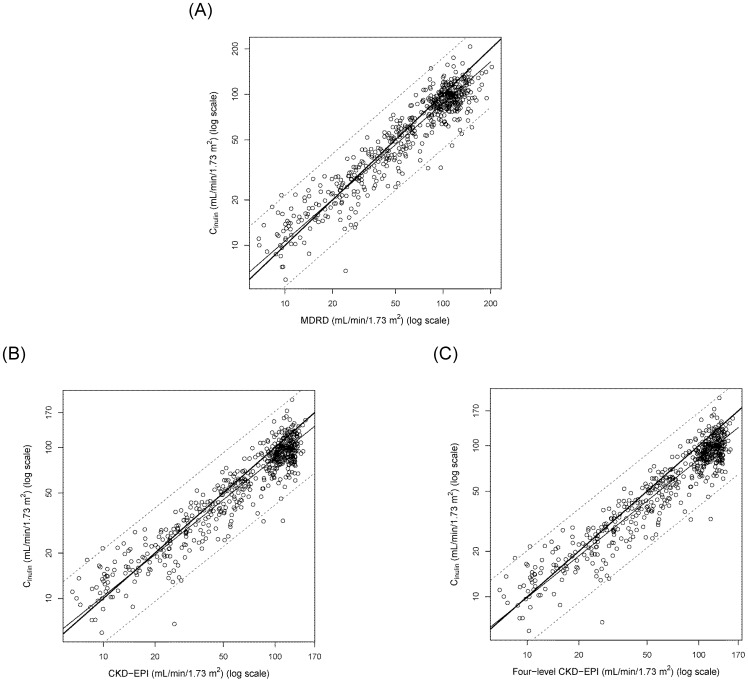
Determination of the Taiwanese eGFR equations in the development set. Taiwanese eGFR equations were derived by using linear regression of the differences on the average (i.e. the extended Bland-Altman plot). C_in_ and the eGFR equations were log-transformed and plotted on the log-scale. The regression line (thin solid line) and its 95% prediction interval (dotted lines) were plotted along with the identity (thick solid diagonal) line. (A) The regression equation of the Taiwanese MDRD equation was 1.309×MDRD^0.912^ in the (anti-logged) original unit. (B) The regression equation of the Taiwanese CKD-EPI equation was 1.262×CKD-EPI^0.914^ in the original unit. (C) The regression equation of the Taiwanese four-level CKD-EPI equation was 1.205×four-level CKD-EPI^0.914^ in the original unit.

Taiwanese eGFR equations were determined by using linear regression of the difference on the average (i.e. the extended Bland-Altman plot) in the development set [22]. Taiwanese eGFR equations were 1.309×MDRD^0.912^ for the Taiwanese MDRD (Fig. 1A), 1.262×CKD-EPI^0.914^ for the Taiwanese CKD-EPI (Fig. 1B) and 1.205×four-level CKD-EPI^0.914^ for the Taiwanese four-level CKD-EPI (Fig. 1C) in the (anti-logged) original units, respectively. In 2,000 bootstraps of the development set, RMSE of the Taiwanese MDRD (11.7) was lower than that of the Taiwanese CKD-EPI (13.7) and Taiwanese four-level CKD-EPI (18.3). Note that RMSE of internal validation was lower than that of the external validation. However, the results of internal validation are usually too optimistic and external validation is necessary for the generalizability of prediction rules [32].

### Accuracy of eGFR for the validation set

Accuracy was assessed by P_30_, bias, precision and the RMSE. We found P_30_ for all of the equations except that of the Japanese equations, Taiwanese CKD-EPI or Taiwanese four-level CKD-EPI equations was lower than that of the Taiwanese MDRD equation for the whole set (Table 2) and those with C_in_≥60 mL/min/1.73 m^2^ (Table3). In contrast, only P_30_ of the four-level CKD-EPI, Asian CKD-EPI and Thai MDRD equations were lower than that of the Taiwanese MDRD equation for those with C_in_<60 mL/min/1.73 m^2^ (Table 4)

**Table 2:** Performance of the eGFR equations for the validation set.

	P_30_ (%)	Bias (mL/min/1.73 m^2^)	Precision (mL/min/1.73 m^2^)	RMSE (mL/min/1.73 m^2^)	Kappa
MDRD	63.3*	−5.4**	23	23.3	0.437
CKD-EPI	60.4**	−8.0**	25	24.2#	0.38
Four-level CKD-EPI	52.5**	−12.0**	29	26.1*	0.38
Japanese MDRD	71.2	5.8**	19	23.7#	0.48
Japanese CKD-EPI	70.5	4.0**	20	23.4	0.494
Asian MDRD	56.8**	−11.0**	28	27.0**	0.435
Asian CKD-EPI	54.0**	−11.0**	28	26.0**	0.38
Thai MDRD	52.5**	−14.0**	32	29.0**	0.435
Taiwanese MDRD	73.4	0.17	19	21.4	0.495
Taiwanese CKD-EPI	73.4	0.42	20	23.0	0.549
Taiwanese four-level CKD-EPI	74.1	0.24	20	23.0	0.549

Abbreviations and definitions: MDRD, Modification of Diet in Renal Disease; CKD-EPI, Chronic Kidney Disease Epidemiology Collaboration; P_30_, the percentage within 30% of inulin clearance; bias, median difference of inulin clearance and estimated GFR (C_in_ – eGFR); precision, interquartile range of the bias; RMSE, root mean square error; Kappa, kappa coefficients of the eGFR equations for the classification of CKD stages.

# P<0.05 versus Taiwanese MDRD;

*p<0.01 versus Taiwanese MDRD;

**P<0.001 versus Taiwanese MDRD.

The Taiwanese equations had the lowest bias among all of the equations for the whole set (Table 2), those with C_in_≥60 mL/min/1.73 m^2^ (Table 3) and those with C_in_<60 mL/min/1.73 m^2^ (Table 4). The Taiwanese and the Japanese equations had the best precision (19–20 mL/min/1.73 m^2^) among all of the equations for the whole set (Table 2). The Taiwanese equations, the Japanese CKD-EPI equation, and the MDRD equation had the lowest RMSE among all equations for the whole set (Table 2). In contrast, the Taiwanese MDRD equation and the Japanese equations had the lowest RMSE for those with C_in_<60 mL/min/1.73 m^2^ (Table 4). However, the Taiwan MDRD equation had lower RMSE than did all equations except the Taiwanese CKD-EPI, Taiwanese four-level CKD-EPI, MDRD and CKD-EPI equations for those with C_in_≥60 mL/min/1.73 m^2^ (Table 3).

**Table 3:** Performance of the eGFR equations for the participants with C_in_≥60 mL/min/1.73 m^2^ in the validation set.

	P_30_ (%)	Bias (mL/min/1.73 m^2^)	Precision (mL/min/1.73 m^2^)	RMSE (mL/min/1.73 m^2^)	Kappa
MDRD	65.0*	−9.2**	30	27.0	0.257
CKD-EPI	65.0*	−12.0**	26	26.7	0.205
Four-level CKD-EPI	57.5**	−16.6**	26	28.4#	0.205
Japanese MDRD	78.8	8.6**	26	27.9#	0.28
Japanese CKD-EPI	81.3	5.2**	26	28.7#	0.29
Asian MDRD	60.0*	−17.0**	30	31.1#	0.26
Asian CKD-EPI	57.5**	−16.0**	26	28.3#	0.26
Thai MDRD	55.0**	−22.0**	31	33.9#	0.26
Taiwanese MDRD	81.3	2.1	28	25.8	0.271
Taiwanese CKD-EPI	81.3	1.2	26	27.5	0.33
Taiwanese four-level CKD-EPI	81.3	1.4	26	27.5	0.33

Abbreviations and definitions: MDRD, Modification of Diet in Renal Disease; CKD-EPI, Chronic Kidney Disease Epidemiology Collaboration; P_30_, the percentage within 30% of inulin clearance; bias, median difference of inulin clearance and estimated GFR (C_in_ – eGFR); precision, interquartile range of the bias; RMSE, root mean square error; Kappa, kappa coefficients of the eGFR equations for the classification of CKD stages.

# P<0.05 versus Taiwanese MDRD;

*p<0.01 versus Taiwanese MDRD;

**P<0.001 versus Taiwanese MDRD.

**Table 4:** Performance of the eGFR equations for the participants with C_in_<60 mL/min/1.73 m^2^ in the validation set.

	P_30_ (%)	Bias (mL/min/1.73 m^2^)	Precision (mL/min/1.73 m^2^)	RMSE (mL/min/1.73 m^2^)	Kappa
MDRD	61.0	−1.7**	17	17.0**	0.356
CKD-EPI	54.0	−3.1**	21	20.3**	0.31
Four-level CKD-EPI	45.8*	−5.0**	24	22.6**	0.31
Japanese MDRD	61.0	3.9**	13	12.3	0.424
Japanese CKD-EPI	56.0	2.4**	15	13.9	0.407
Asian MDRD	52.5	−4.8**	21	20.2**	0.365
Asian CKD-EPI	49.0#	−5.1**	24	22.4**	0.291
Thai MDRD	49.0*	−5.6**	23	21.9**	0.365
Taiwanese MDRD	62.7	−0.58	15	13.2	0.429
Taiwanese CKD-EPI	62.7	−0.61	15	14.7*	0.411
Taiwanese four-level CKD-EPI	64.4	−0.62	15	14.8*	0.411

Abbreviations and definitions: MDRD, Modification of Diet in Renal Disease; CKD-EPI, Chronic Kidney Disease Epidemiology Collaboration; P_30_, the percentage within 30% of inulin clearance; bias, median difference of inulin clearance and estimated GFR (C_in_ – eGFR); precision, interquartile range of the bias; RMSE, root mean square error; Kappa, kappa coefficients of the eGFR equations for the classification of CKD stages.

# P<0.05 versus Taiwanese MDRD;

*p<0.01 versus Taiwanese MDRD;

**P<0.001 versus Taiwanese MDRD.

### Agreement between C_in_ and eGFR for the validation set

The agreement between C_in_ and the eGFR equations was assessed by concordance correlation and the Bland-Altman plot for the validation set. We found that concordance correlation of the Taiwanese MDRD equation (0.823) was higher (P<0.05) than those of the Japanese MDRD (0.803), Taiwanese CKD-EPI (0.78), Taiwanese four-level CKD-EPI (0.78), Thai MDRD (0.772) and Japanese CKD-EPI (0.771) equations, but was not different from that of the MDRD (0.826), CKD-EPI (0.798), Asian MDRD (0.794), Asian CKD-EPI (0.782),or four-level CKD-EPI (0.781), for the whole set.

The concordance correlation of the Taiwanese MDRD equation (0.757) was higher (P<0.05) than those of the Taiwanese CKD-EPI (0.729), Taiwanese four-level CKD-EPI (0.728), MDRD (0.685), CKD-EPI (0.628), Asian MDRD (0.627), Asian CKD-EPI (0.593), four-level CKD-EPI (0.59) and Thai MDRD (0.598) equations, but was not different from that of the Japanese MDRD (0.771) or Japanese CKD-EPI (0.746) equation, for those with C_in_<60 mL/min/1.73 m^2^.

The concordance correlation of the Taiwanese MDRD (0.537) was higher (P<0.05) than those of the Taiwanese four-level CKD-EPI (0.39) and Taiwanese CKD-EPI (0.388) and Japanese CKD-EPI (0.378) equations, but was not different from that of the MDRD (0.571), Asian MDRD (0.522), Japanese MDRD (0.502), Thai MDRD (0.489), CKD-EPI (0.464), four-level CKD-EPI (0.447) or Asian CKD-EPI (0.446), for those with C_in_≥60 mL/min/1.73 m^2^.

**Figure 2:**
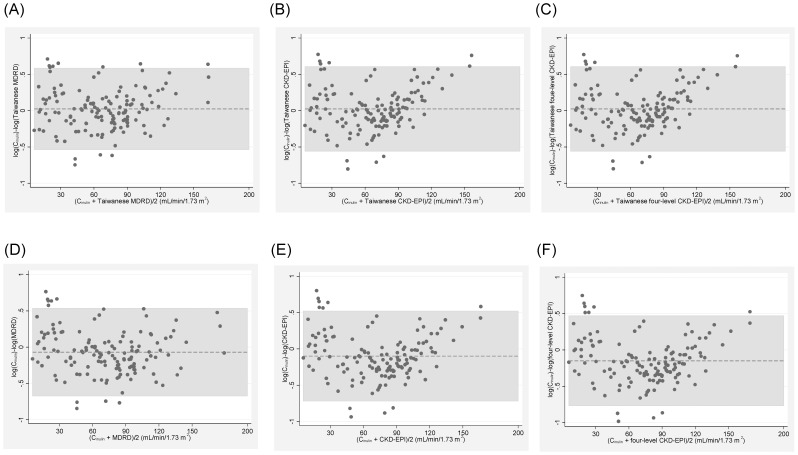
Bland-Altman plot of eGFR equations versus inulin clearance (C_in_) for the validation set. The difference of log-transformed C_in_ and eGFR equation (ordinate) was plotted against the mean of C_in_ and each respective eGFR equation (abscissa). The mean difference was shown as a dotted horizontal line whereas the 95% limit of agreement was shown as the shaded area. Note that the anti-log of the difference between two log-transformed data is the ratio of the two data in the original units. Bland-Altman plot of (A) The geometric mean ratio (95% limit of agreement) was 1.023 (0.58, 1.79) for the Taiwanese MDRD in the original unit, (B) The geometric mean ratio (95% limit of agreement) was 1.025 (0.57, 1.8) for the Taiwanese CKD-EPI in the original unit, (C) The geometric mean ratio (95% limit of agreement) was 1.025 (0.57, 1.8) for the Taiwanese four-level CKD-EPI, (D) The geometric mean ratio (95% limit of agreement) was 0.93 (0.5, 1.7) for the MDRD in the original unit, (E) The geometric mean ratio (95% limit of agreement) was 0.91 (0.49, 1.7) for the CKD-EPI in the original unit and (F) The geometric mean ratio (95% limit of agreement) was 0.86 (0.46, 1.6) for the four-level CKD-EPI in the original unit.

In the Bland-Altman plot of the original data (data not shown), there were increasing scatters of differences with increasing eGFR (i.e. V-shaped limits of agreement) for all of the equations. Moreover, there were negative correlations between the difference and the mean for the MDRD, CKD-EPI and four-level CKD-EPI equations respectively. These findings violated the assumptions of the homogeneity of the scatter of differences and a constant bias for the Bland-Altman plot [22], [26], [33]. Thus, the Bland-Altman plot of the difference of the log-transformed data on the ordinate [33] was shown in Fig. 2. Note that the anti-log of the difference between two log-transformed data is the ratio of the two data in the original units. The geometric mean ratio (95% limit of agreement) in the original units of the Taiwanese MDRD (A), Taiwanese CKD-EPI (B), Taiwanese four-level CKD-EPI (C), MDRD (D), CKD-EPI (E) and four-level CKD-EPI (F) equations was 1.023 (0.58, 1.79), 1.025 (0.57, 1.8), 1.025 (0.57, 1.8), 0.93 (0.5, 1.7), 0.91 (0.49, 1.7), 0.86 (0.46, 1.6), respectively.

**Figure 3:**
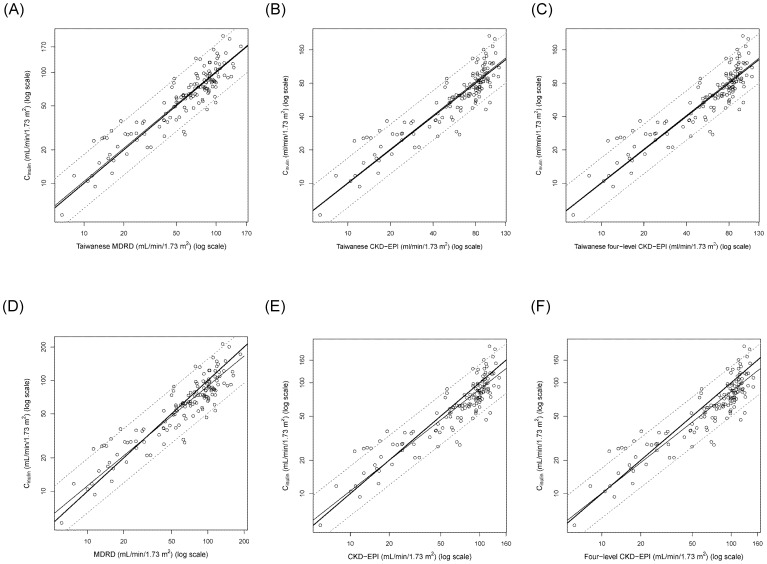
Regression of the Taiwanese eGFR equations versus inulin clearance (C_in_) in the validation set. C_in_ and the eGFR equations were log-transformed and plotted on the log-scale in the regression derived from the extended Bland-Altman plot. The regression line (thin solid line) and its 95% prediction interval (dotted lines) were plotted along with the identity (thick solid diagonal) line. (A) The slope (95% confidence interval) was 0.99 (0.92, 1.06) and the regression equation of the Taiwanese MDRD was 1.06×Taiwanese MDRD^0.99^ in the anti-logged (original) unit. (B) The slope was 1.008 (0.93, 1.08) and the regression equation of the Taiwanese CKD-EPI was 0.99×Taiwanese CKD-EPI^1.01^ in the original unit. (C) The slope was 1.007 (0.93, 1.08) and the regression equation of the Taiwanese four-level CKD-EPI was 0.99×Taiwanese four-level CKD-EPI^1.01^ in the original unit. (D) The slope was 0.9 (0.83, 0.96) and the regression equation of the MDRD equation was 1.41×MDRD^0.9^ in the original unit. (E) The slope was 0.92 (0.85, 0.99) and the regression equation of the CKD-EPI equation was 1.28×CKD-EPI^0.92^ in the original unit. (F) The slope was 0.92 (0.85, 0.99) and the regression equation of the four-level CKD-EPI was 1.22×four-level CKD-EPI^0.92^ in the original unit.

Regression lines and the 95% prediction intervals of the extended Bland-Altman plot of the log-transformed C_in_ and the log-transformed Taiwanese MDRD (Fig. 3A), Taiwanese CKD-EPI (Fig. 3B), Taiwanese four-level CKD-EPI (Fig. 3C), MDRD (Fig. 3D), CKD-EPI (Fig. 3E) and four-level CKD-EPI (Fig. 3F) in the (anti-logged) original units are shown in Fig. 3.

The slope (95% confidence interval) of the Taiwanese MDRD, Taiwanese CKD-EPI, Taiwanese four-level CKD-EPI, MDRD, CKD-EPI, four-level CKD-EPI, Japanese MDRD, Japanese CKD-EPI, Asian MDRD, Asian CKD-EPI and Thai MDRD was 0.99 (0.92, 1.06), 1.008 (0.93, 1.08), 1.007 (0.93, 1.08), 0.9 (0.83, 0.96), 0.92 (0.85, 0.99), 0.92 (0.85, 0.99), 0.9 (0.83, 0.96), 0.92 (0.85, 0.99), 0.9 (0.83, 0.96), 0.92 (0.85, 0.99) and 0.9 (0.83, 0.96), respectively. Note that only the Taiwanese equations had no proportional bias (i.e. they had slopes that were not different from 1).

### Diagnostic performances of the eGFR equations for the classification of CKD stages for the validation set

Diagnostic performance of the eGFR equations for the classification of CKD stages was assessed by the ordinal logistic regression and the kappa coefficients [28]. We found that all the eGFR equations had similar kappa values compared with that of the Taiwanese MDRD equation for the whole set (Table 2), those with C_in_≥60 mL/min/1.73 m^2^ (Table 3) and those with C_in_<60 mL/min/1.73 m^2^ (Table 4).

In ordinal logistic regression, the Taiwanese MDRD equation had the lowest AIC (230.7) compared to those of the MDRD (231.4), CKD-EPI (232), four-level CKD-EPI (232.2), Taiwanese CKD-EPI (231.4), Taiwanese four-level CKD-EPI (231.5), Japanese MDRD (231.4), Japanese CKD-EPI (232), Asian MDRD (231.4), Asian CKD-EPI (232) and Thai MDRD (231.4) equations. Moreover, the Taiwanese MDRD equation had the highest Akaike weight (0.14) compared to those of the MDRD (0.097), CKD-EPI (0.07), four-level CKD-EPI (0.07), Taiwanese CKD-EPI (0.098), Taiwanese four-level CKD-EPI (0.09), Japanese MDRD (0.097), Japanese CKD-EPI (0.07), Asian MDRD (0.097), Asian CKD-EPI (0.07) and Thai MDRD (0.097) equations. Thus, the Taiwanese MDRD equation had the best diagnostic performance.

## Discussion

In this study, the Taiwanese equations had the lowest bias, the Taiwanese equations and the Japanese CKD-EPI equation had the lowest RMSE, whereas the Taiwanese and the Japanese equations had the best precision and the highest P_30_. The Taiwanese MDRD equation had higher concordance correlation than the Taiwanese CKD-EPI, Taiwanese four-level CKD-EPI and the Japanese equations. Moreover, only the Taiwanese equations had no proportional bias among all of the equations. Finally, the Taiwanese MDRD equation had the best diagnostic performance in terms of ordinal logistic regression among all of the equations.

We found that the MDRD, CKD-EPI, four-level CKD-EPI, Asian equations, and Thai MDRD equations overestimated GFR, whereas the Japanese equations underestimated GFR. In contrast, the Taiwanese equations had very low bias in that the Taiwanese equations had the lowest bias among all equations.

The Taiwanese and the Japanese equations had similar performances in terms of P_30_ and precision in that they had the highest P_30_ and the best precision among all equations. Moreover, the Taiwanese equations and the Japanese CKD-EPI equation had the lowest RMSE among all equations. However, the Taiwanese equations had lower biases than those of the Japanese equations and the Taiwanese MDRD equation had lower RMSE than those of the Japanese equations for those with C_in_≥60 mL/min/1.73 m^2^. Note that P_30_ is an arbitrary measure of accuracy whereas dichotomization (i.e., P_30_) of continuous variables introduces biases [34]. In contrast, bias and RMSE are standard measures of model accuracy [35]. Moreover, the Taiwanese MDRD equation had higher concordance correlation than the Japanese equations. Finally, the Japanese, but not the Taiwanese equations, had proportional bias. Thus, the Taiwanese MDRD equation is better than the Japanese equations.

In the Bland-Altman plot, the 95% limits of agreement were too wide to draw conclusions. Nonetheless, only the Taiwanese equations had no proportional bias among all equations in the extended Bland-Altman plot [22].

Surprisingly, the Taiwanese MDRD equation had similar kappa value with the other equations. This finding can be explained by the fact that eGFR is a continuous variable and that the eGFR-defined CKD stage is an arbitrary categorical variable whereas dichotomization of continuous predictor variables introduces biases [34]. In contrast, the Taiwanese MDRD equation-derived eGFR (a continuous variable) had the best diagnostic performance in terms of the lowest AIC and the highest Akaike weight in the ordinal logistic regression.

Many (but not all) studies found that the CKD-EPI equation was more accurate than the MDRD equation [6], [24], [36]-[38]. For example, the Japanese CKD-EPI equation was better than the Japanese MDRD equation [10]. The CKD-EPI equation was also better than the MDRD and four-level CKD-EPI equations in a Chinese study [39]. However, the CKD-EPI equation was not better than the MDRD study equation for diabetic patients [37], [38]. In this study, the Taiwanese CKD-EPI and Taiwanese four-level CKD-EPI equations were worse than the Taiwanese MDRD equation especially for those with C_in_<60 mL/min/1.73 m^2^

The differences in ethnicity, study population and the use of different reference GFR methods may account for this discrepancy. For example, our study and the Japanese study [10] used the gold standard C_in_ as the reference GFR, whereas the MDRD and CKD-EPI equation used urinary clearance of iothalamate, which overestimates C_in_ [40]-[42]. In contrast, the Singapore and the Thai studies [11], [20] used plasma technetium-^99m^-labeled diethylenetriamine penta-acetate (^99m^Tc-DTPA) clearance, which also overestimates C_in_ [43], [44].

The strengths of this study were the use of the gold standard (C_in_) as the reference GFR, the inclusion of healthy volunteers, and the validation of the Taiwanese eGFR equations using an external validation set. One of the limitations of this study was that there were no healthy volunteers in the validation set. However, the mean C_in_ was similar and the proportion of subjects with C_in_<60 mL/min/1.73 m^2^ was also similar between the development and the validation set. The other limitation was that the participants were older in the external validation set. However, differences in case mix is not a great concern for external validation [31]. Finally, both hospitals used the same study protocol and serum creatinine and inulin were both measured at the Kaohsiung Medical University Hospital.

In conclusion, the Taiwanese MDRD equation performs better than the MDRD, CKD-EPI, four-level CKD-EPI, Japanese, Asian, Thai, Taiwanese CKD-EPI, and Taiwanese four-level CKD-EPI equations for Taiwanese adults. Thus, further studies are required to determine its clinical applications (e.g. correlations with complications and prognosis) in Taiwan.

## Supporting Information

Figure S1 **Logistic regression for C_in_-defined CKD stage 1 in the validation set.** Linear regression (the oblique line) and logistic regression (the S-shaped sigmoid curve) of the Taiwanese MDRD equation for the prediction of the probability (π(*x*)) of inulin clearance (C_in_)-defined CKD stage 1 (the dots on the vertical axis, yes = 1, no = 0). Note that the probability of CKD stage 1 increases as eGFR increases and that the logistic curve always predicts π(*x*) to be within the limit of zero and 1 whereas the linear regression line can predict π(*x*) to be less than zero.
(TIFF)

Figure S2 **Ordinal logistic regression of the Taiwanese MDRD equation for the prediction of C_in_-defined CKD stages in the validation set.** Ordinal logistic regression (generalized ordered logit) of the Taiwanese MDRD equation was performed by the cumulative logit model. (A) Cumulative probability of C_in_-defined CKD stages and the logistic curves. Note that the cumulative probability of CKD stage 1, stage 1–2, stage 1–3 and stage 1–4 increases as eGFR increases. (B) Taiwanese MDRD equation was used to predict the log(odds) (logit) of the probability of CKD stage 1, stage 1–2, stage 1–3 and stage 1–4. Note that the non-linear relationship between cumulative probability and eGFR in (A) had been transformed to be a linear (α+βx) relationship. The odds ratio (95% confidence interval) of one unit increase in x was calculated as exp(β), which was 1.09 (1.05, 1.13), 1.11 (1.07, 1.15), 1.14 (1.08, 1.21) and 2.01 (1.06, 3.82) for stage 1, stage 1–2, stage 1–3 and stage 1–4, respectively.
(TIFF)

Methods S1 **Supplemental methods-ordinal logistic regression.**
(DOC)
