# Supplementary figures and images for: Modification of Diet in Renal Disease (MDRD) Study and CKD Epidemiology Collaboration (CKD-EPI) Equations for Taiwanese Adults
Source: PLoS One. 2014 Jun 13;9(6):e99645. doi: 10.1371/journal.pone.0099645 (PMC4057229; doi:10.1371/journal.pone.0099645)

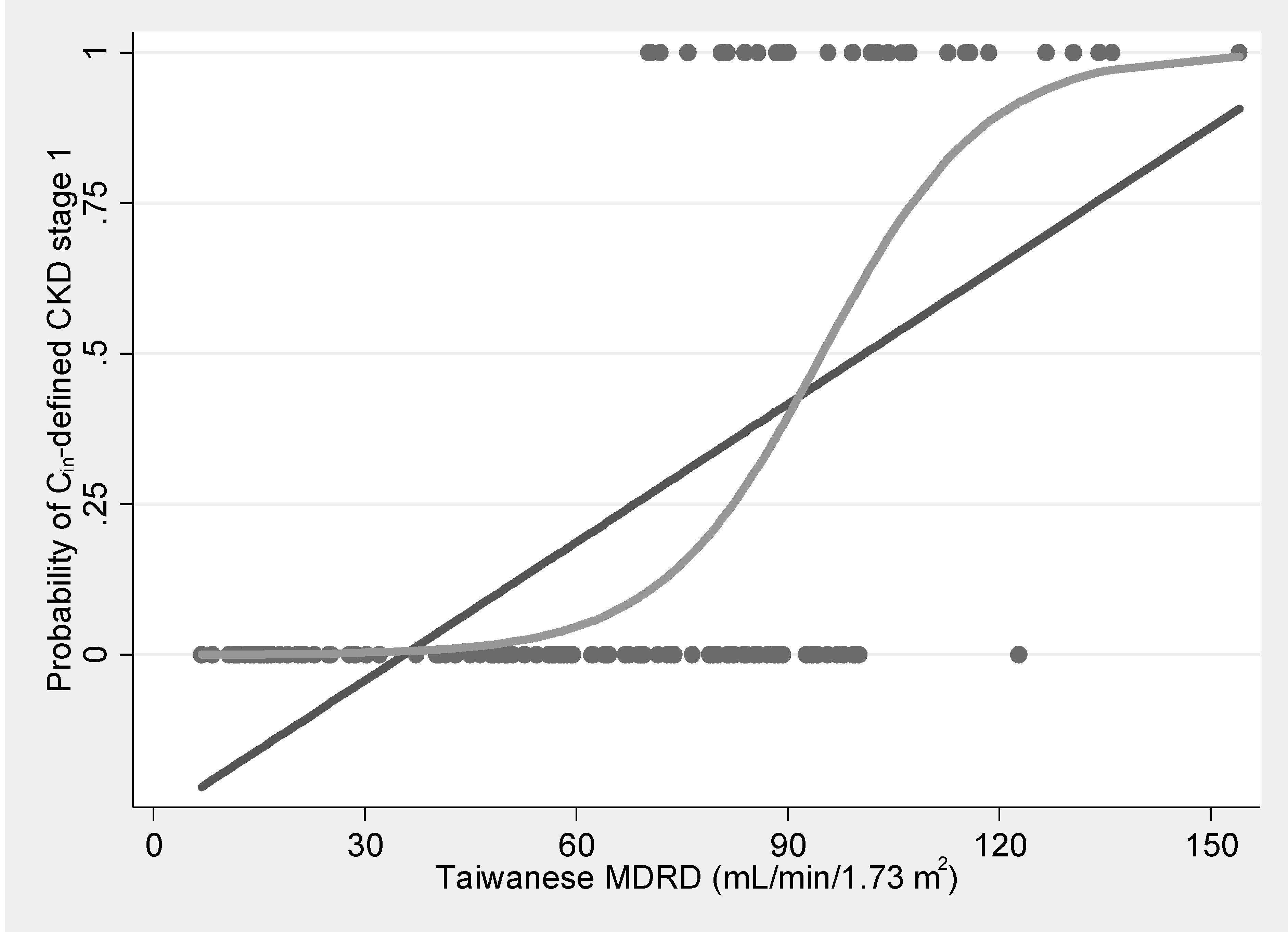

Supplement: Figure S1 — Logistic regression for Cin-defined CKD stage 1 in the validation set. Linear regression (the oblique line) and logistic regression (the S-shaped sigmoid curve) of the Taiwanese MDRD equation for the prediction of the probability (π(x)) of inulin clearance (Cin)-defined CKD stage 1 (the dots on the vertical axis, yes = 1, no = 0). Note that the probability of CKD stage 1 increases as eGFR increases and that the logistic curve always predicts π(x) to be within the limit of zero and 1 whereas the linear regression line can predict π(x) to be less than zero. (TIFF) [file pone.0099645.s001.tif]

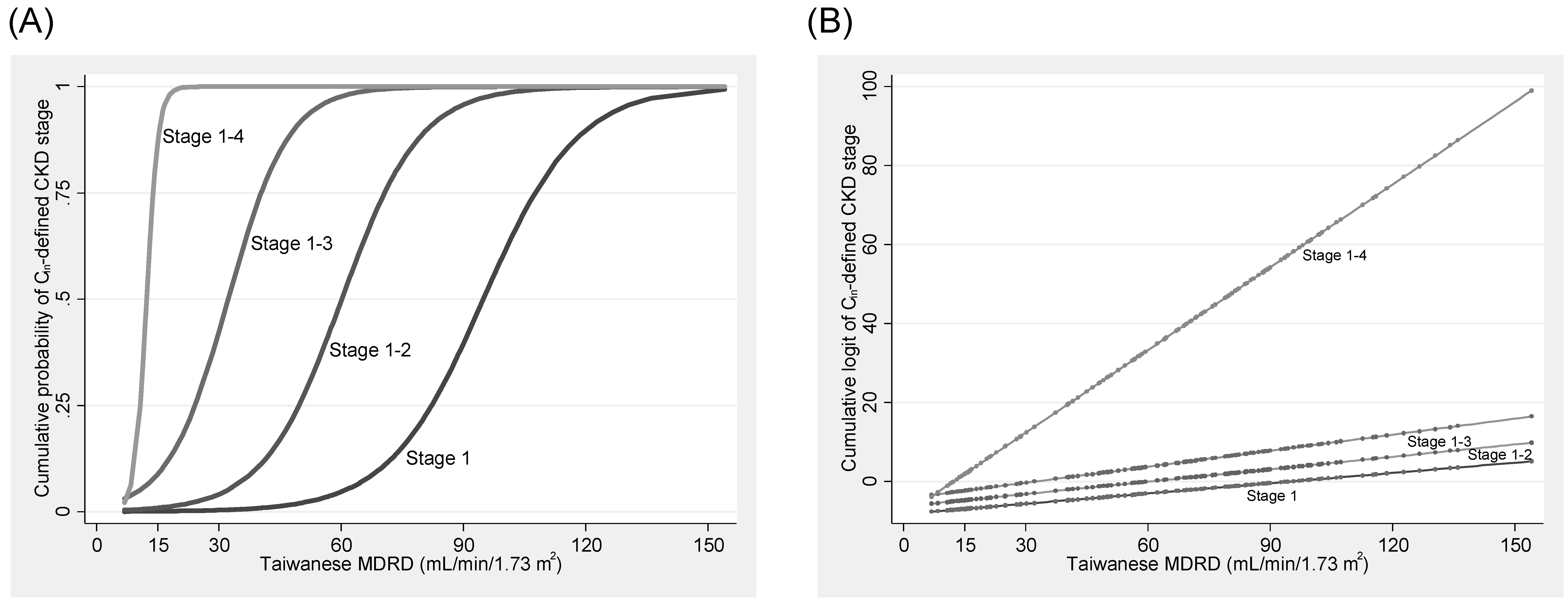

Supplement: Figure S2 — Ordinal logistic regression of the Taiwanese MDRD equation for the prediction of Cin-defined CKD stages in the validation set. Ordinal logistic regression (generalized ordered logit) of the Taiwanese MDRD equation was performed by the cumulative logit model. (A) Cumulative probability of Cin-defined CKD stages and the logistic curves. Note that the cumulative probability of CKD stage 1, stage 1–2, stage 1–3 and stage 1–4 increases as eGFR increases. (B) Taiwanese MDRD equation was used to predict the log(odds) (logit) of the probability of CKD stage 1, stage 1–2, stage 1–3 and stage 1–4. Note that the non-linear relationship between cumulative probability and eGFR in (A) had been transformed to be a linear (α+βx) relationship. The odds ratio (95% confidence interval) of one unit increase in x was calculated as exp(β), which was 1.09 (1.05, 1.13), 1.11 (1.07, 1.15), 1.14 (1.08, 1.21) and 2.01 (1.06, 3.82) for stage 1, stage 1–2, stage 1–3 and stage 1–4, respectively. (TIFF) [file pone.0099645.s002.tif]
