# Supplementary material for: Modification of Diet in Renal Disease (MDRD) Study and CKD Epidemiology Collaboration (CKD-EPI) Equations for Taiwanese Adults
Source: PLoS One. 2014 Jun 13;9(6):e99645. doi: 10.1371/journal.pone.0099645 (PMC4057229; doi:10.1371/journal.pone.0099645)
Supplement: Methods S1 — Supplemental methods-ordinal logistic regression. (DOC) [file pone.0099645.s003.doc]

**Supplemental methods-ordinal logistic regression**

Logistic regression is useful for classification. For a binary outcome (Y, 0=no, 1=yes) with a binomial distribution, the probability is the average of Y. The conditional probability of Y given *x* (the independent variable) is: P(*Y* = 1$\left|X=x\right.$) = π(*x*) and P(*Y* = 0$\left|X=x\right.$) = 1- π(*x*), which are bound between 0 and 1. We may use linear regression (a simple linear model where the parameters are estimated by the ordinary least squares) to predict π(*x*) from *x*: π(*x*) = α + β*x* (e.g. Fig. S1). However, this model is not adequate because *x* can take any value so that there is no guarantee that π(*x*) will be between 0 and 1 unless the coefficients are severely restricted [1-2]. A solution is to non-linearly transform the probability by the logit transformation to remove the restrictions [1-2]. The logit of π(*x*) is defined as log ($\frac{\text{ }\pi\text{(x)}}{1-\pi(x)}$) = log(odds). Logistic regression is a generalized linear model for binary outcomes with binomial distribution where the link is the logit [1-3]. For simplicity, this supplement only discusses the models with a single independent variable.

The logistic function is π(*x*) = $\frac{\exp(\alpha+\beta x)}{1+\exp(\alpha+\beta x)}$ = $\frac{odds}{1+odds}$ where *x* is the independent variable, exp is the exponential. Logit[π(*x*)] = log(odds) = log ($\frac{\pi(x)}{1-\pi(x)}$) = α + β*x* where α is the intercept, β (the parameter) is the log-odds ratio of one unit increase in *x* whereas exp(β) is the odds ratio of one unit increase in *x*. Note that the logistic function is a non-linear function of *x* whereas α + β*x* is a linear function of *x* (Fig. S1).

Note that odds has no ceiling but has a floor of zero (i.e. it ranges from 0 to ∞). So we use log(odds) which has no ceiling or floor to accommodate the possible range of α + β*x* (-∞ ~ +∞). In logistic regression, the regression coefficients (βs) are estimated by using the maximum likelihood method which identifies the regression coefficients that maximize the log likelihood (the likelihood of the parameter given the observed outcomes) by an iterative calculation [1, 2].

The ordinal logistic regression is used for the ordinal outcomes. The ordinal logistic regression often uses the cumulative logit models [3, 4]. Consider an ordinal outcome variable Y with *k* categorical outcomes, denoted by *j*=1,…, *k*. The probability P(Y = *j*) = π*_j_*, the probability P(Y ≤ *k*) = π*_k_* = 1 and the *k*-1 cumulative probability P(Y ≤ *j*) = π*_1_* +…+ π*_j_* for j=1,…, *k*-1 [3, 4]. A cumulative logit is defined as

logit [P(Y ≤ *j*)] = log$\left[\frac{P(Y\leq j)}{1-P(Y\leq j)}\right]$ = log$\left[\frac{P(Y\leq j)}{P(Y>j)}\right]$ = log$\left[\frac{\pi_{1}+...+\pi_{j}}{\pi_{j+1}+...+\pi_{k}}\right]$

The cumulative logit reflects the ordering, with logit [P(Y ≤ 1)] ≤ logit [P(Y ≤ 2)] ≤ logit [P(Y ≤ *k*-1)]. A cumulative logit model looks like an ordinary logistic model in which categories 1 to *j* combine to form a single (lower) category, and categories *j*+1 to *k* combine to form a second (higher) category [3, 4]. For example (Fig. S2A), CKD stage 1 was a single category and stage 2-5 was a second category. CKD stages 1 and 2 combined to form a single category and CKD stage 3-5 was a second category. CKD stages 1 to 3 combined to form a single category and CKD stage 4-5 was a second category. CKD stages 1 to 4 combined to form a single category and CKD stage 5 was a second category.

The cumulative logit model consists of the proportional odds model and the generalized ordered logit model. In the proportional odds (parallel lines) model, β does not dependent on *j*. In other words, the log-odds ratios (βs) are identical across *k* outcomes. In contrast, the intercept (α_j_) varies across *k* outcomes.

P(Y ≤ *j*|*x*) = $\frac{\exp(\alpha_{j}+\beta x)}{1+\exp(\alpha_{j}+\beta x)}$

Cumulative logit of the lower category = log$\left[\frac{P(Y\leq j\left|x)\right.}{P(Y>j\left|x)\right.}\right]$ = $\alpha_{j}+\beta x$

where P(Y ≤ *j*|*x*) is the cumulative probability of the event Y ≤ *j* given *x* and P(Y > *j*|*x*) is the cumulative probability of the event Y > *j* given *x*, β is the log(odds ratio) and exp(β) is the cumulative odds ratio of being in the lower rather than the higher half of the dichotomy [3].

In the generalized ordered logit model, the assumption of proportional odds is not required and β varies in different *j* categories [4, 5].

P(Y ≤ *j*|*x*) = $\frac{\exp(\alpha_{j}+\beta_{j}x)}{1+\exp(\alpha_{j}+\beta_{j}x)}$

log$\left[\frac{P(Y\leq j\left|x)\right.}{P(Y>j\left|x)\right.}\right]$ = $\alpha_{j}+\beta_{j}x$

The prevalence of C_in_-defined CKD stage 1, 2, 3, 4 and 5 was 23.74%, 33.81%, 22.3%, 15.83% and 4.32% in the validation set, respectively. Thus, the cumulative prevalence of C_in_-defined CKD stage 1-2, stage 1-3 and stage 1-4 was 57.55%, 79.85% and 95.68%, respectively. The ordinal logistic regression-predicted average probability of stage 1, 2, 3, 4 and 5 for the Taiwanese MDRD equation was 23.98%, 33.91%, 22.35%, 15.61% and 4.15%, respectively. Thus, the average cumulative probability of CKD stage 1-2, stage 1-3 and stage 1-4 for the Taiwanese MDRD equation was 57.89%, 80.24% and 95.85%, respectively. Ordinal logistic regression performed by the cumulative logit model of CKD stage 1, stage 1-2, stage 1-3 and stage 1-4 for the Taiwanese MDRD equation was shown in Fig. S2A and Fig. S2B.

For the Taiwanese MDRD equation, the odds ratio for CKD stage ≤ 1 versus stage > 1 (i.e. stage 2-5) was 1.09. In other words, for each unit increase in eGFR, the odds of being in stage 1 is 1.09-fold of the odds of being in stage 2-5. On the other hand, the odds ratio for stage ≤ 2 (i.e. stage 1-2) versus stage > 2 (i.e. stage 3-5) was 1.11. In other words, for each unit increase in eGFR, the odds of being in stage 1-2 is 1.11-fold of the odds of being in stage 3-5. Finally, the odds ratio for stage 1-3 (stage ≤ 3) versus stage 4-5 (stage >3) was 1.14, and for stage 1-4 (stage ≤ 4) versus stage 5 (stage > 4) was 2.01.

Note that the Taiwanese MDRD equation is only used as an example of the ordinal logistic regression. The results of the ordinal logistic regression for the other eGFR equations can be calculated similar to this example whereas the relative performances of the ordinal logistic regressions for the various eGFR equations should be compared by the AIC and the Akaike weight (*wi*).

**References**

1. Dominguez-Almendros S, Benitez-Parejo N, Gonzalez-Ramirez AR (2011) Logistic regression models. Allergologia et immunopathologia 39: 295-305.

2. Tripepi G, Jager KJ, Dekker FW, Zoccali C (2008) Linear and logistic regression analysis. Kidney Int 73: 806-810.

3. Agresti A (2002) Logit Models for Multinomial Responses. in: Agresti A ed: Categorical Data Analysis (2^nd^ ed.). p. 267-313. Hoboken, New Jersey: John Wiley & Sons, Inc.

4. Ananth CV, Kleinbaum DG (1997) Regression models for ordinal responses: a review of methods and applications. International journal of epidemiology 26: 1323-1333.

5. Fu VK (1999) Estimating generalized ordered logit models. Stata Technical Bulletin 8: 27-30.
